# Associations between fatty acid oxidation, hepatic mitochondrial function, and plasma acylcarnitine levels in mice

**DOI:** 10.1186/s12986-018-0241-7

**Published:** 2018-01-29

**Authors:** Bodil Bjørndal, Eva Katrine Alterås, Carine Lindquist, Asbjørn Svardal, Jon Skorve, Rolf K. Berge

**Affiliations:** 1 Department of Clinical Science, University of Bergen, N-5020 Bergen, Norway; 2 Department of Heart Disease, Haukeland University Hospital, N-5021 Bergen, Norway

**Keywords:** Beta-oxidation, Mitochondrial function, Oxidative phosphorylation, Tetradecylthioacetic acid, Tetradecylthiopropionic acid, Acylcarnitine

## Abstract

**Background:**

The 4-thia fatty acid tetradecylthiopropionic acid (TTP) is known to inhibit mitochondrial β-oxidation, and can be used as chemically induced hepatic steatosis-model in rodents, while 3-thia fatty acid tetradecylthioacetic acid (TTA) stimulates fatty acid oxidation through activation of peroxisome proliferator activated receptor alpha (PPARα). We wished to determine how these two compounds affected in vivo respiration and mitochondrial efficiency, with an additional goal to elucidate whether mitochondrial function is reflected in plasma acylcarnitine levels.

**Methods:**

C57BL/6 mice were divided in 4 groups of 10 mice and fed a control low-fat diet, low-fat diets with 0.4% (*w*/w) TTP, 0.4% TTA or a combination of these two fatty acids for three weeks (*n* = 10). At sacrifice, β-oxidation and oxidative phosphorylation (OXPHOS) capacity was analysed in fresh liver samples. Hepatic mitochondria were studied using transmission electron microscopy. Lipid classes were measured in plasma, heart and liver, acylcarnitines were measured in plasma, and gene expression was measured in liver.

**Results:**

The TTP diet resulted in hepatic lipid accumulation, plasma L-carnitine and acetylcarnitine depletion and elevated palmitoylcarnitine and non-esterified fatty acid levels. No significant lipid accumulation was observed in heart. The TTA supplement resulted in enhanced hepatic β-oxidation, accompanied by an increased level of acetylcarnitine and palmitoylcarnitine in plasma. Analysis of mitochondrial respiration showed that TTP reduced oxidative phosphorylation, while TTA increased the maximum respiratory capacity of the electron transport system. Combined treatment with TTP and TTA resulted in a profound stimulation of genes involved in the PPAR-response and L-carnitine metabolism, and partly prevented triacylglycerol accumulation in the liver concomitant with increased peroxisomal β-oxidation and depletion of plasma acetylcarnitines. Despite an increased number of mitochondria in the liver of TTA + TTP fed mice, the OXPHOS capacity was significantly reduced.

**Conclusion:**

This study indicates that fatty acid β-oxidation directly affects mitochondrial respiratory capacity in liver. As plasma acylcarnitines reflected the reduced mitochondrial β-oxidation in TTP-fed mice, they could be useful tools to monitor mitochondrial function. As mitochondrial dysfunction is a major determinant of metabolic disease, this supports their use as plasma markers of cardiovascular risk in humans. Results however indicate that high PPAR activation obscures the interpretation of plasma acylcarnitine levels.

**Electronic supplementary material:**

The online version of this article (10.1186/s12986-018-0241-7) contains supplementary material, which is available to authorized users.

## Background

Mitochondrial dysfunction is implicated in the development of many metabolic diseases such as metabolic syndrome, non-alcoholic fatty liver disease (NAFLD) and type 2 diabetes mellitus (T2DM), conditions which are highly linked to increased risk of cardiovascular disease (CVD) and myocardial infarction (MI) [1–4]. The mitochondria have several crucial roles in energy metabolism, including the Krebs cycle, ketone body production, ATP production through oxidative phosphorylation of the respiratory chain (OXPHOS), and β-oxidation of fatty acids. Fatty acids (C > 14) cannot translocate into the mitochondria, hence they are transported through the carnitine shuttle; Fatty acids are linked to Coenzyme A (fatty acyl-CoA) in the cytosol by acyl-CoA synthetases. Membrane bound carnitine palmitoyltransferase 1 (CPT1) then exchanges CoA with a carnitine molecule, producing fatty acylcarnitine. Acylcarnitine passes the inner mitochondrial membrane into the matrix through a carnitine transporter, where CPT2 exchanges the carnitine molecule with CoA. The acyl-CoA is then ready for β-oxidation. Inhibition of fatty acid β-oxidation is prominent in mitochondrial dysfunction, preventing the removal of excess lipids in the cells, which can result in an increase in toxic intermediate lipid products, including acylcarnitines [5]. In line with this, plasma levels of long-chain acylcarnitines are highly linked to increased mortality in chronic heart failure patients [6]. In obese individuals, overloaded mitochondria will increase the presence of toxic lipid intermediates that can potentially damage the mitochondria, while the removal of excess lipids by increased fat burning is considered beneficial [7]. NAFLD is defined by accumulation of lipids in the liver (mainly in the form of triacylglycerol (TAG)) exceeding 5% per liver weight in the absence of significant alcohol consumption [8]. With a prevalence exceeding 15% in the general population and even higher in people that are overweight, obese or have diabetes, NAFLD is considered one of the most common liver disease in the world [9]. Although it is not an indicator of metabolic syndrome, NAFLD is associated with obesity, insulin resistance and dyslipidemia [10, 11] and can be considered an early predictor of metabolic syndrome [12]. Recently, metabolomics analysis has strongly indicated that defects in lipid regulation are established prior to hyperglycemia and T2DM [13]. Despite its high prevalence, no approved treatments have been defined for NAFLD besides alteration in diet and increased exercise. Treatments targeting mitochondrial function have the potential to ameliorate NAFLD and thus prevent CVD and T2DM.

Artificially generated fatty acid analogues with a sulphur atom included in the carbon chain (thia-fatty acids) are useful tools to investigate the effect of β-oxidation on mitochondrial function and plasma CVD risk factors in animals. Tetradecylthiopropionic acid (TTP), with sulphur in the fourth position from the carboxylic end, has been demonstrated to inhibit mitochondrial β-oxidation [14]. TTP is transported into mitochondria by carnitine palmitoyltransferase 1 and 2 (CPT1 and CPT2), and oxidized to TTAcr-CoA (tetradecylthioacrylic-coenzyme A). This metabolite will accumulate since it is a relatively poor substrate for mitochondrial hydratase and CPT2, resulting in inhibition of CPT2, and thus inhibition of fatty acid oxidation [15]. As a consequence, treatment with TTP increases hepatic TAG levels and induces fatty liver in rats [15–20]. Tetradecylthioacetic acid (TTA), with sulphur in the third position from the carboxylic end, is on the other hand an activator of mitochondrial and peroxisomal β-oxidation [14, 18–21], through its role as a pan-PPAR ligand [19, 22–24]. High-fat fed rats treated with TTA for 50 weeks had lower levels of plasma acylcarnitines compared to control [25]. TTA has also been shown to reduce body weight, adipose tissue depots, and plasma and hepatic TAG levels in rodent studies [26].

In this study, we investigated how hepatic mitochondrial respiration, plasma acylcarnitine levels and lipid levels in plasma, liver and heart were affected by TTP- and TTA-supplemented diets for three weeks in male C57BL/6 mice. The aim was to gain more knowledge on how mitochondrial β-oxidation affects mitochondrial OXPHOS capacity and CVD risk factors. A secondary aim was to use TTP-treatment as a model of chemically induced hepatic steatosis, and investigate whether co-treatment with TTA would prevent TTP-induced hepatic lipid accumulation.

## Methods

### Animals and diets

The animal study was conducted in accordance with the Guide for the Care and Use of Laboratory Animals. The protocol was approved by the Norwegian State Board of Biological Experiments with Living Animals (Project no. 5071). Male C57BL/6JBomTac mice, between 8 and 10 weeks old, were purchased from Taconic (Ry, Denmark). Upon arrival, the mice were placed in open cages, two in each cage, and they were allowed to acclimatize to their surroundings for one week before starting the experiment. The mice were kept in a 12 h light/dark cycle at a constant temperature (22 ± 2 °C) and a relative humidity of 55% (± 5%). During the acclimatization period the mice had unrestricted access to chow and tap water. The cages were block-randomized to four different diets, 10 mice per diet, equally distributed between four days of feeding start/sacrifice: Group 1 (control) was given a low-fat diet consisting of 7% (*w*/w) fat from lard (5% w/w) and soy bean oil (2% w/w) purchased from MP Biomedicals (Illkirch France) and Dyet Inc. (Bethlehem, PA, USA) respectively. The feed also contained 19% (*w*/w), casein and cornstarch, dyetrose, sucrose, fiber, AIN-93-MX mineral mix, AIN-93-VX vitamin mix, L-cysteine, choline bitartrate (Dyets inc.), and tert-Butyl-hydroquinone purchased from Sigma Aldrich [27]. Groups 2–4 were given low-fat diets supplemented with 0.4% (w/w) TTP, 0.4% (w/w) TTA or a combination of 0.4% (w/w) TTP and 0.4% (w/w) TTA. Both TTA and TTP were prepared at the Department of Chemistry, University of Bergen [28]. Mice were fed a fixed amount (9 g dry weight per two mice) daily for 21 days.

At sacrifice, mice were fasted for 4 h, and anaesthetized by inhalation of 2% isoflurane (Schering-Plough, Kent, UK). The abdomen was opened in the midline and EDTA-blood was collected by cardiac puncture and immediately chilled on ice. The samples were centrifuged and plasma was stored at − 80 °C prior to analysis. Heart, liver, and adipose tissues (epididymal, perirenal, and subcutaneous white adipose tissue depots) were collected and weighed. Fresh samples from each liver was removed for β-oxidation analysis and respiratory analysis, one sample was fixed for transmission electron microscopy (TEM), while the heart and the remaining parts of the liver were immediately snap-frozen in liquid nitrogen and stored at − 80 °C until further analysis.

### Quantification of plasma, liver and heart parameters

Liver and heart lipids were extracted from frozen samples according to Bligh and Dyer [29], evaporated under nitrogen, and redissolved in isopropanol before analysis. Lipids from liver, heart and plasma were measured enzymatically on a Hitachi 917 system (Roche Diagnostics GmbH, Mannheim, Germany) using the cholesterol (Cholesterol CHOD-PAP, 11,491,458–216), and triacylglycerol (Triglycerides GPO-PAP, 11,730,711) kit from Roche Diagnostics, and the free cholesterol (Free Cholesterol FS, Ref 113,609,910,930), non-esterified fatty acid (NEFA FS, Ref 157,819,910,935) and phospholipid kit (Phospholipids FS, Ref 157,419,910,930) from DiaSys (Diagnostic Systems GmbH, Holzheim, Germany). L-carnitine, trimethyl-lysine, γ-butyrobetaine, palmitoylcarnitine and acetylcarnitine were analysed in plasma samples, some pooled from 2 to 3 animals (*n* = 3–6), by HPLC-MS/MS as described by Vernez et al. [30] with some modifications [31].

### Histology

Cryo-sections from frozen liver samples were generated using a 1720 Cryostat (Leica Microsystems, Wetzlar, Germany) from 3 to 4 mice per group. The cry-sections were fixed in 4% buffered formalin for 10 min, rinsed 3× in dH_2_O, and stained in 0.7% (*w*/*v*) Oil Red O (Sigma) in propylene glycol for 10 min, rinsed 3× dH_2_O, and stained with hematoxylin (Thermo Fisher Scientific, Waltham, MA, USA) for 2 min. Finally, sections were rinsed 3× dH_2_O and mounted with ImmuMount (Thermo Fisher Scientific). Images were captured using an Olympus BX51 light microscope at 40× magnification with an Olympus DP25 digital colour camera (Olympus Corporation, Tokyo, Japan). Three images per section were captured by a blinded investigator, and representative images were selected.

### Hepatic enzyme activities

A 250 mg liver sample was chilled on ice and homogenized in 1 mL ice-cold sucrose medium (0.25 M sucrose, 10 mM HEPES, and 1 mM Na_4_EDTA, adjusted to a pH of 7.4 with KOH). The homogenates were centrifuged at 860 G for 10 min at 4°C and the post-nuclear fraction was removed and used for further analysis. β-oxidation capacity was measured as previously described, using (1-^14^C) palmitoyl CoA as substrate [32, 33]. The post-nuclear fraction was frozen at − 80 °C and later used to measure Acyl-CoA oxidase 1, palmitoyl (ACOX1) activity as described by Madsen et al. [34], with some modifications [35].

### Mitochondrial respiratory capacity

An 8 mg sample of liver was harvested from 4 to 6 mice per group and kept on ice in cold BIOPS solution for 15–30 min, before homogenization in cold Mir05buffer using a PSI-Shredder HHR-Set (Oroboros Instruments, Innsbruck, Austria). Buffers were made according to http://bioblast.at/index.php/MiPNet03.02_Chemicals-Media. The homogenate was divided between to two chambers of an Oroboros Oxygraph-2 k instrument. Samples from 2 animals were run in parallel, using 4 chambers in total per run. The mitochondrial respiratory capacity analysis was carried out according to the substrate-uncoupler-inhibitor titration (SUIT) protocol described by Pesta and Gnaiger [36] with a few changes. Activators and inhibitors were added in the order and concentration given in Table 1. Maximum mitochondrial capacity was measured by stepwise addition of 1 mM carbonyl cyanide p-(trifluoro-methoxy) phenyl-hydrazone (FCCP), 10 ul per step, until maximum oxygen consumption was achieved.

**Table 1:** Addition of substrate and inhibitors

Compound	End concentration	Purpose
Malate	0.5 mM	Supplies complex I with substrate
Octanoylcarnitine	0.2 mM	Supplies ETF with electrons
ADP	2.5 mM	To assess oxidative phosphorylation
Glutamate	10 mM	Supplies complex I with substrate
Succinate	10 mM	Supplies complex II with substrates
FCCP		Uncouples ETS from oxidative phosphorylation
Rotenone	0.5 μM	Inhibits the electron flux from the Fe-S centre to Q in complex I
Antimycin A	2.5 mM	Inhibits the electron flux from heme bH to Q in complex III, to observe non-mitochondrial respiration.

### Hepatic gene expression

Total cellular RNA was purified from 20 mg frozen liver samples, and cDNA was produced as described by Vigerust et al. [37]. Real-time PCR was performed with Sarstedt 384-well Multiply-PCR plates (Sarstedt Inc., Newton, NC, USA) on the following genes, using probes and primers from Applied Biosystems (Foster City, CA, USA): acyl-coenzyme A oxidase 1, palmitoyl (*Acox1* Mm01246834_m), gamma-butyrobetaine hydroxylase 1 (*Bbox1* Mm00499905_m1), CD36 antigen/fatty acid translocase (*Cd36*/*Fat* Mm00432403_m1), carnitine palmitoyltransferase 1a and 2 (*Cpt1a* Mm01231183_m1 and *Cpt2* Mm00487255_m1, respectively), carnitine acetyltransferase (*Crat* Mm00483985_m1), fatty acid binding protein 1, liver (*Fabp1*, Mm00444340-m1), NADH dehydrogenase (ubiquinone) Fe-S protein 1 (*Ndufs1,* Mm00523640_m1), succinate dehydrogenase complex, subunit A, flavoprotein (*Sdha,* Mm01352366_m1), uncoupling protein 2 (*Ucp2*, Mm00627599_m1).

Three different reference genes were used; Eukaryotic 18S ribosomal RNA (*18S*, Kit-FAM-TAMRA (Reference RT-CKFT-18 s) from Eurogentec, Belgium), glyceraldehyde-3-phosphate dehydrogenase (*Gapdh*, Mm99999915_g1, from Applied Biosystems), Ribosomal protein, large, P0 (*Rplp0*, Gene ID 11837, from Thermo Fisher Scientific). The optimal normalization gene was determined using Normfinder [38], and data normalized to *Rplp0* are presented.

### Transmission electron microscopy

Fresh liver tissue was cut into approximately 1 mm cubes and fixed in 2.5% glutaraldehyde in 0.1 M sodium cacodylate buffer at 4 °C for 4–5 days before further processing. After being washed twice in 0.1 M sodium cacodylate buffer on the shaker for 1 h, samples were post fixated for 1 h in 200 μl freshly made osmium tetraoxide in 0.1 M sodium cacodylate, washed twice in 0.1 M sodium cacodylate buffer, 5 min each, and dehydrated using increasing ethanol concentration (30% ethanol 10 min, 50% ethanol 15 min, 2 × 70% ethanol). After 1–4 weeks, samples were further dehydrated in 96% ethanol (2 × 10 min) and 100% ethanol (2 × 20 min) before propylene oxide (1,2 epoxy propane) was added and left for approximately 20 min. Epoxy resin was added gradually over a period of about an hour before it was removed. The tubes were filled about half way up with epoxy resin and left for an hour with the caps off to let residue propylene oxide evaporate. Finally, liver samples were added to labelled trays, filled with epoxy resin and left overnight before the epoxy resin was polymerized at 60 °C for 48 h. Samples were sectioned and stained with uranyl acetate and lead citrate by the Molecular Imaging Center at the University of Bergen (www.uib.no/en/rg/mic), and the samples were magnified using Joel JEM-1230 transmission electron microscope (Joel Ltd., Tokyo, Japan). Images of three chosen histological sections of each sample were acquired with a GATAN multiscan camera (Gatan Inc., Pleasanton, CA, USA). The images were captured at a magnification of 5000, 10,000 and 25,000, approximately 3 images per mouse. The image collection was partly blinded as the images of hepatic lipid droplets indicated TTP treatment. The number of mitochondria was estimated from samples from 4 mice.

### Statistical analysis

Data was analysed using Prism Software (Graph-Pad Software, San Diego, CA) to determine statistical significance. The results are shown as means of 4–10 animals per group with their standard deviations. When possible, normal distribution was determined by the Kolmogorov-Smirnov test (with Dallal-Wilkinson-Lilliefor *P* value). One-way ANOVA with Dunnet’s post hoc test was used to evaluate statistical differences between groups. *P*-values < 0.05 were considered statistically significant.

## Results

### Effect on body, adipose tissue and liver weights

**Fig. 1:**
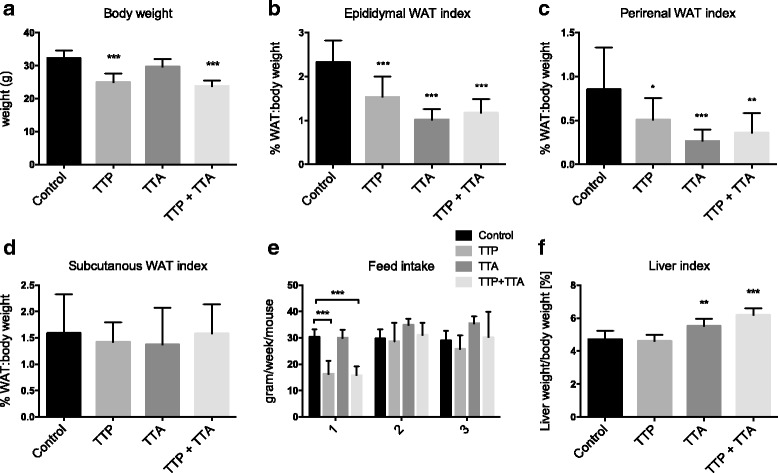
Weight gain and organ weights in male C57/BL6 mice fed 0.4% (*w*/w) TTP, 0.4% (w/w) TTA, or TTP + TTA for 3 weeks. **a** Final body weight, (**b**) index of epididymal white adipose tissue (WAT weight/body weight*100), (**c**) index of perirenal WAT, (**d**) index of subcutaneus WAT, (**e**) weekly feed intake per mouse, and (**f**) liver index (liver weight/body weight*100). Values are means with standard deviations (*n* = 8–10). Significant difference from controls was determined using one-way ANOVA with Dunnett’s post hoc test, comparing treatment groups to the control group (**p ≤ 0.01, ***p ≤ 0.001)

C57BL/6 mice were fed low-fat diets containing 0.4% (*w*/w) TTP, 0.4% (w/w) TTA, or a combination of the two thia-fatty acid analogues for 3 weeks. The body weight was lower in both groups fed TTP (Fig. 1a) accompanied with a significant reduction of white adipose tissue mass i.e. epididymal and perirenal fat depots (data not shown), as well as the percentage adipose tissue:body weight (Fig. 1b and c). The subcutaneous fat pat indexes were not affected by any of the diets (Fig. 1d). It was of interest to note that TTA also reduced the epididymal and perirenal adipose index without changing the body weight. The daily feed intake in week 2 and 3 was not changed by the thia fatty acid analogues, although TTP-fed mice demonstrated a lower the feed intake in week 1 (Fig. 1e). In the TTA and TTP + TTA group the percentage liver:body weight was significantly increased compared to control (Fig. 1f).

### Hepatic fatty acid oxidation and plasma carnitine esters

**Fig. 2:**
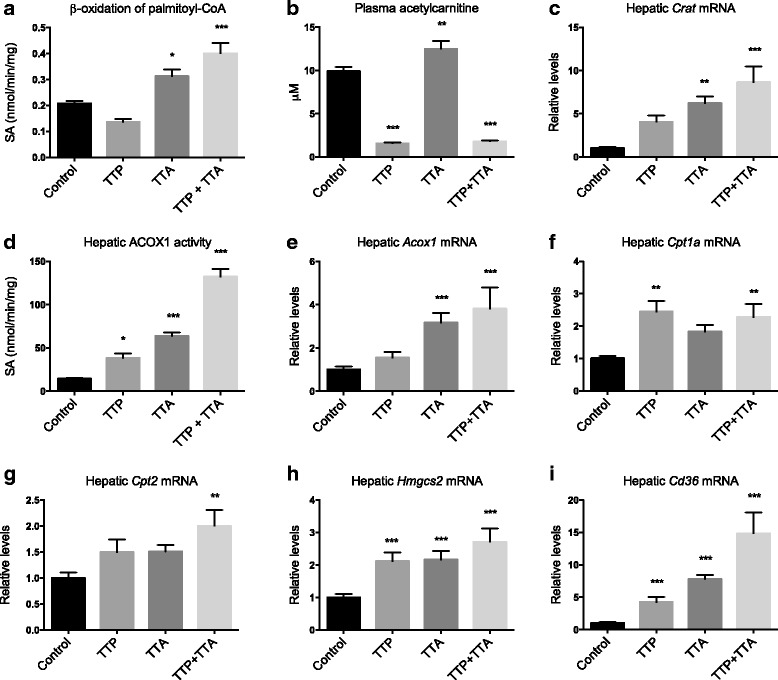
Liver enzyme activity in male C57/BL6 mice fed 0.4% (w/w) TTP, 0.4% (w/w) TTA, or TTP + TTA for 3 weeks. **a** β-oxidation of palmitoyl-Coenzyme A (CoA) (*n* = 6), (**b**) plasma acetylcarnitine (*n* = 3–6), (**c**) hepatic *Crat* gene expression, (**d**) hepatic acyl-CoA oxidase (ACOX) activity (*n* = 6), (**e**) hepatic *Acox1* gene expression, (**f**) hepatic *Cpt1* gene expression, (**g**) hepatic *Cpt2* gene expression, (**h**) hepatic *Hmgcs2* gene expression, and (**i**) hepatic *Cd36* gene expression. Values are means with standard deviations (*n* = 8 unless otherwise stated). Significant difference from controls was determined using one-way ANOVA with Dunnett’s post hoc test (*p ≤ 0.05, **p ≤ 0.01, ***p ≤ 0.001)

The β-oxidation of palmitoyl-CoA was measured *in vitro* in fresh liver homogenates, and demonstrated an increased fatty acid catabolism in TTA-fed mice (Fig. 2a). This was accompanied by increased plasma levels of acetylcarnitine, an end product of mitochondrial fatty acid oxidation (Fig. 2b). Moreover, the TTA administration significantly increased the hepatic gene expression of *Crat*, encoding a mitochondrial matrix enzyme carnitine acetyltransferase that transfers short acyl groups from acyl-CoA to L-carnitine, thus regulating the acyl-CoA/CoA ratio of the cells (Fig. 2c). TTP is reported to inhibit the mitochondrial fatty acid oxidation [14]. In this experiment TTP tended to reduce the *in vitro* palmitoyl-CoA oxidation, but the data were not significant at *p* < 0.05. However, the plasma acetylcarnitine level was significantly reduced by TTP treatment while the gene expression of *Crat* was unchanged (Fig. 2b,c). Interestingly, in liver homogenates from mice treated with both TTA and TTP, *in vitro* palmitoyl-CoA oxidation was highly increased and the gene expression of *Crat* upregulated whereas the plasma level of acetylcarnitine was decreased (Fig. 2a-c).

As TTP specifically inhibits mitochondrial β-oxidation at the CPT2-step [15], we wished to determine the contribution from peroxisomal β-oxidation. Hepatic ACOX1 activity was significantly increased in both TTP and TTA-fed mice, and a synergistic effect was observed in co-treated mice (Fig. 2d). The gene expression of *Acox1* in liver was increased in the TTA and TTP + TTA group (Fig. 2e). The gene expression of *Cpt1a* and *2* were significantly increased by TTP + TTA, and *Cpt1a* was significantly increased by TTP (Fig. 2f,g). Another PPAR-response gene, *Hmgcs2*, the mitochondrial enzyme in ketone body production, was significantly increased in liver by all three interventions (Fig. 2h). There were no significant differences in the gene expression of fatty acid binding protein *(Fabp1)* in liver (data not shown). However, there was a significant increase in the gene expression of *Cd36* in all treatment groups, and results indicate a synergistic effect on the uptake of free fatty acids in the co-treatment group (Fig. 2i).

**Fig. 3:**
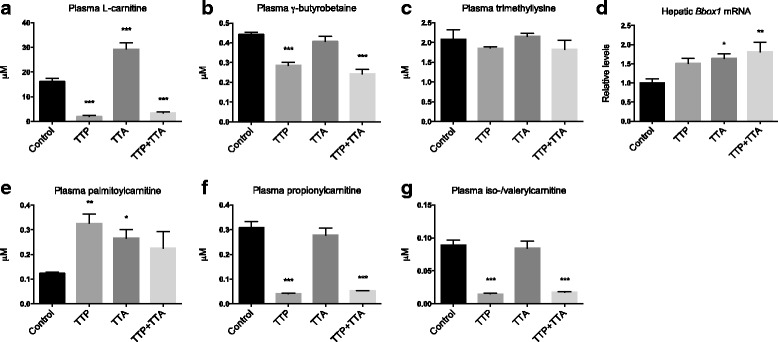
Plasma L-carnitine, carnitine percursors and acylcarnitines in male C57/BL6 mice fed 0.4% (w/w) TTP, 0.4% (w/w) TTA, or TTP + TTA for 3 weeks. **a** L-carnitine, (**b**) trimethyllysine, (**c**) g-butyrobetaine, (**d**) hepatic *Bbox1* gene expression (*n* = 8), (**e**) palmitoylcarnitine, (**f**) propionylcarnitine, and (**g**) iso−/L-valerylcarnitine. Values are means with standard deviations (*n* = 3–6, unless otherwise stated). Significant difference from controls was determined using one-way ANOVA with Dunnett’s post hoc test (*p ≤ 0.05, **p ≤ 0.01, ***p ≤ 0.001)

As mentioned above, long-chain fatty acids have to be esterified to L-carnitine to be transported in and out of the mitochondria. Plasma levels of unesterified L-carnitine were significantly reduced by TTP and TTP + TTA (8.2-fold and 4.7-fold reduction, respectively) (Fig. 3a). In addition, the direct carnitine precursor γ-butyrobetaine was reduced, while trimethyllysine was unaffected by TTP (Fig. 3b,c). TTA on the other hand increased the L-carnitine plasma level compared to control without affecting its precursors. This was linked to an increase in the gene expression of *Bbox1*, responsible for the generation of L-carnitine from γ-butyrobetaine (Fig. 3d). A synergistic increase in *Bbox1* expression was observed in the TTP + TTA-group, despite the decrease in free L-carnitine in this group. In contrast, the 16-carbon chain palmitoylcarnitine was elevated in plasma by TTP and TTA, but only insignificantly by TTP + TTA (Fig. 3e). Propionylcarnitine and iso−/valerylcarnitine, products of branched chain and amino acid degradation, were also reduced by TTP and TTP + TTA (Fig. 3f,g). Octanoylcarnitine, an intermediate product of β-oxidation, was not increased by any of the diets (data not shown).

### Influence of TTP and TTA on tissue lipid levels

**Fig. 4:**
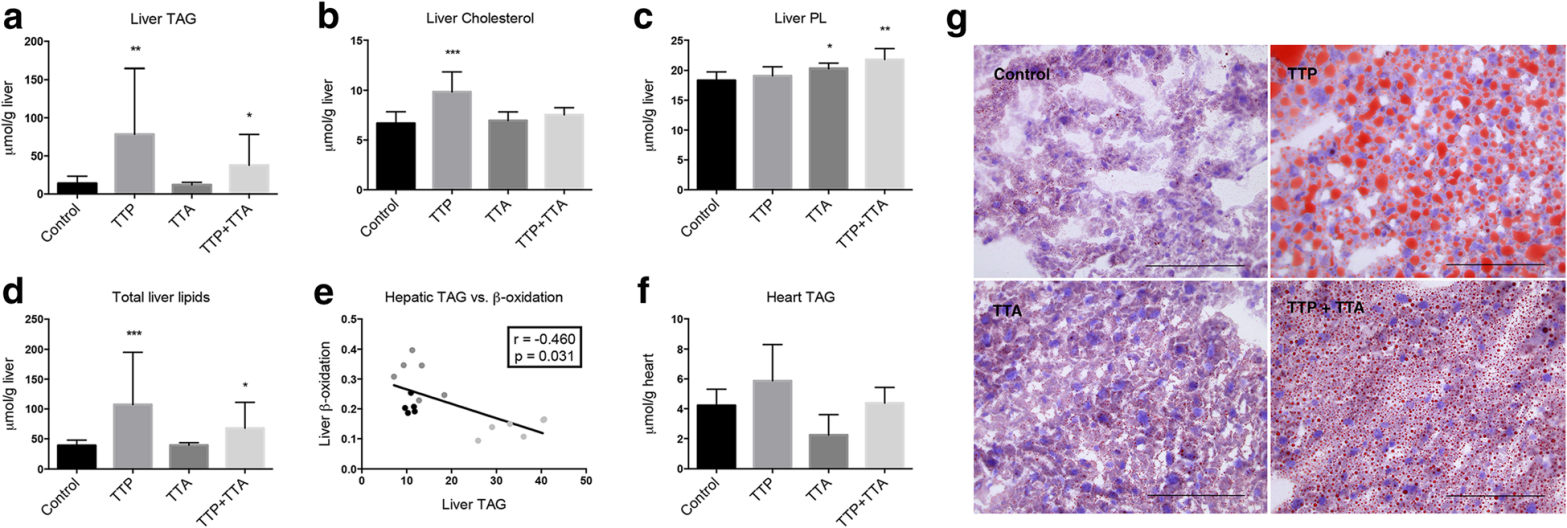
Tissue lipid levels in male C57/BL6 mice fed 0.4% (w/w) TTP, 0.4% (w/w) TTA, or TTP + TTA for 3 weeks. **a** Hepatic triacylglycerol (TAG), (**b**) hepatic cholesterol, (**c**) hepatic phospholipids, (**d**) total hepatic lipids, (**e**) linear regression of liver TAG and β-oxidation of palmitoyl-coenzyme A (*n* = 18, black circles - control, light gray circles – TTP, grey circles - TTA), (**f**) heart TAG (*n* = 5). Values are means with standard deviations (*n* = 8, unless otherwise stated). Significant difference from controls was determined using one-way ANOVA with Dunnett’s post hoc test (*p ≤ 0.05, **p ≤ 0.01, ***p ≤ 0.001). **g** Representative images of liver cryo-sections stained with Oil Red O and captured at 40× magnification using a light microscope (*n* = 3–4). The black line indicates 100 μm

Although *in vitro* β-oxidation was not significantly reduced by TTP, a 7.3 fold increase in hepatic TAG was observed compared to control (Fig. 4a). Hepatic cholesterol was also increased, while phospholipids were unchanged, resulting in a 2.8-fold increase in total lipids in liver by TTP (Fig. 4b-d). TTA did not affect liver lipid levels, with the exception of a small, but significant increase in phospholipids. The accumulation of TAG and total liver lipids in the TTP + TTA co-treated mice tended to be of less magnitude than in mice fed TTP alone (1.7-fold and 2.2-fold increase compared to control, respectively). A weak but significant negative correlation was observed between *in vitro* palmitoyl-CoA oxidation in liver homogenates and hepatic TAG concentration in the control, TTP and TTA group (Fig. 4e, r = -0.460, *p* = 0.031). In contrast to findings in liver, TAG levels in heart tissue were not significantly affected by thia-fatty acid diets (Fig. 4f). The size of liver lipid droplets was greatly enhanced in TTP-treated mice compared to control (Fig. 4g). TTA and TTP co-treatment was able to reduce the size of lipid droplets compared to TTP alone, but they remained larger than in livers of control- and TTA-treated animals.

### Influence of TTP and TTA on plasma lipids

The efficiency of mitochondrial β-oxidation can strongly influence plasma parameters linked to risk evaluation in CVD. In this study, despite their opposite effect on mitochondrial β-oxidation, both TTP and TTA led to a reduction of plasma TAG levels and phospholipid levels (Table 2). Each thia-fatty acid alone did not influence plasma total cholesterol. However, the co-treatment increased total cholesterol. Strikingly, TTP increased plasma non-esterified fatty acids compared to control, and this was not seen in TTP + TTA-fed mice.

**Table 2:** Plasma lipid classes in mice treated for 3 weeks with a control low-fat diet, or low-fat diets supplemented with 0.4% (w/w) TTP, TTA or TTP + TTA

Plasma levels (mmol/L)^1^	Diet groups			
	Control	TTP	TTA	TTP + TTA
TAG	1.29 ± 0.28	0.54 ± 0.27***	0.66 ± 0.22***	0.34 ± 0.11**
Total cholesterol	3.70 ± 0.38	3.39 ± 0.19	3.81 ± 0.25	4.16 ± 0.56*
Phospholipids	3.53 ± 0.26	2.84 ± 0.24***	3.15 ± 0.30*	2.97 ± 0.33**
NEFA	0.17 ± 0.05	0.31 ± 0.18*	0.11 ± 0.05	0.17 ± 0.06

^1^Values are means with standard deviation (*n* = 3–6). Values statistically different from control were determined by one-way ANOVA with Dunnett’s post hoc test. *p < 0.05, **p < 0.01, ***p < 0.001

Abbreviations: *NEFA* Non-esterified fatty acids, *TAG* Triacylglycerol, *TTA* Tetradecylthioacetic acid, *TTP* Tetradecylthiopropionic acid

### Mitochondrial morphology and respiration in liver

**Fig. 5:**
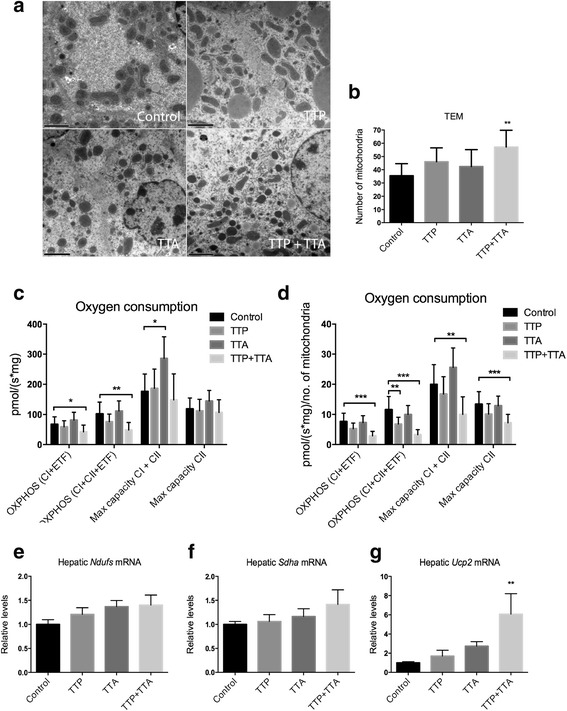
Transmission electron microscopy (TEM) and respiration in liver samples from male C57/BL6 mice fed 0.4% (w/w) TTP, 0.4% (w/w) TTA, or TTP + TTA for 3 weeks. **a** Representative images from TEM, 10,000 x magnification and, (**b**) estimation of number of mitochondria from 3 to 4 TEM images per animal (*n* = 4). **c** Oxygen consumption at the different stages in the SUIT protocol (*n* = 4-6). **d** Oxygen consumption divided by the average number of mitochondria in the different treatment groups (*n* = 4–6). **e** Hepatic gene expression of *Ndufs*, (**f**) *Sdha*, and (**g**) *Ucp2* (*n* = 8). Values are means with standard deviations. Significant difference from controls was determined using one-way ANOVA with Dunnett’s post hoc test (*p ≤ 0.05, **p ≤ 0.01, ***p ≤ 0.001)

The effect of TTP and TTA treatment on mitochondria was further investigated by transmission electron microscopy (TEM) on liver samples. Representative images are shown in Fig. 5a, demonstrating the typical lipid droplets in TTP-treated mice. The average number of mitochondria was estimated from TEM images, and the level was significantly increased in livers from TTP + TTA treated mice (Fig. 5b).

Upon sacrifice, fresh liver samples were harvested and used to determine oxidative phosphorylation (OXPHOS), based on oxygen consumption after addition of octanoylcarnitine and activators of complex I (CI) and II of the respiratory chain in the presence of ADP. Maximum OXPHOS capacity in uncoupled mitochondria was also determined by adding FCCP. TTA increased the maximum capacity compared to control, while TTP + TTA reduced OXPHOS at both CI + ETF and CI + CII + ETF (Fig. 5c). Adjusting results for the number of mitochondria in each treatment group revealed that TTP + TTA treatment strongly reduced OXPHOS at CI + electron transferring flavoprotein (ETS) (Fig. 5d), although no change was observed in the expression of one of the complex I genes, *Ndufs* (Fig. 5e). Both TTP alone and TTP + TTA treatment reduced OXPHOS at CI + CII + ETF compared to control (Fig. 5d), while no significant change was observed in the expression of the complex II gene *Sdha* (Fig. 5f). TTP + TTA significantly reduced maximal capacity in uncoupled mitochondria, and also reduced the maximal capacity when CI was inhibited by rotenone. This was accompanied by a significant increase in the gene expression of uncoupling protein 2 (*Ucp2*), a gene that has been implicated in the oxidative stress response [39] (Fig. 5g). In contrast, TTA stimulated maximum OXPHOS capacity compared to control (Fig. 5c), without a significant effect on *Ucp2* expression (Fig. 5f).

## Discussion

The rapid removal of TAG and NEFAs from circulation, through both β-oxidation of fatty acids for energy production and storage in adipose tissue, is important to prevent metabolic disease and the development of NAFLD. In this study, we analysed how reduction and stimulation of β-oxidation by the 4-thia fatty acid analogue TTP and the 3-thia fatty acid analogue TTA, respectively, influenced hepatic lipid accumulation and plasma levels of lipid metabolites involved in CVD-risk, as well as hepatic mitochondrial function. We also investigated whether co-treatment with TTA could prevent the negative effects of TTP.

The analogues metabolism is grossly similar to the endogenous fatty acids and they are activated to CoA esters. However, while TTA is blocked for ß-oxidation, TTP is oxidized to TTAcr-CoA which is an inhibitor of CPT2. We found elevated TAG levels (Fig. 4a), Oil Red O-stained and TEM-images indicated a distinct accumulation of lipid droplets in the liver of TTP-fed mice (Figs. 4g and 5a), and this was accompanied by decreased plasma acetylcarnitine levels (Fig. 2b). This is in line with a pilot study in rats, where 14 days of TTP treatment gave lipid droplet accumulation in a dose-dependent manner, as seen by Oil Red O staining [40]. This suggests reduced hepatic β-oxidation capacity even though the *in vitro* catabolism of palmitoyl-CoA in liver homogenates only tended to be reduced compared to control (Fig. 2a). In contrast, peroxisomal β-oxidation was increased by TTP at the enzyme activity level. This increase indicates a specific inhibitory effect of TTP on mitochondrial β-oxidation, in agreement with results from previous studies in rats [15, 41, 42].

The hepatic gene expression of the carnitine transferase *Cpt1a* was highly increased by TTP, and this could indicate a compensatory effect to inhibited CPT2 activity. In addition, TTP increased expression of the PPARα-genes *Cd36* and *Hmgcs2*, involved in fatty acid import and keton body production, respectively. This agrees with *in vitro* assays, which have demonstrated that both TTP and TTA are ligands for PPARα, although with different potencies [43]. As expected, TTA treatment significantly increased both mitochondrial and peroxisomal β-oxidation, seen by both enzyme activities and gene expression of *Acox1* (Fig. 2). Also, the liver index increased in TTA-treated mice, a known response to PPARα agonists in rodents. Interestingly, a synergistic effect on enzyme activity and expression of a number of PPARα genes, including *Acox1*, was observed in livers from TTP + TTA-treated mice. Thus, the PPARα effect on ACOX1 activity by TTA seemed to be potentiated by TTP co-treatment. It is possible that the increase in *in vitro* palmitoyl-CoA β-oxidation observed in the TTP + TTA treated mice could partly be due to peroxisomal β-oxidation, and that this effect was sufficient to lower hepatic lipid accumulation and lipid droplet size (Figs. 2 and 4). Mitochondrial β-oxidation may still have been partly blocked as plasma acetylcarnitine, reflecting the end product of mitochondrial β-oxidation, acetyl-CoA, was strongly reduced both in TTP and TTP + TTA treated mice. In support of this, a significant negative correlation between *in vitro* fatty acid oxidation of palmitoyl-CoA and hepatic TAG concentration was observed, but only in control, TTA and TTP animals. The possibility that the active TTP-metabolite (TTAcr-CoA) could have been diluted in the β-oxidation assay should also be considered, thus the inhibitory effect in the presence of TTP may have been stronger in vivo.

Interestingly, despite similar tendencies, the liver was more affected than heart by TTP-induced lipid accumulation (Fig. 4), although mitochondrial fatty acid oxidation is important in both tissues. This sensitivity of the liver towards lipid accumulation may explain the early manifestation of NAFLD during the metabolic syndrome, and our study shows that reduced mitochondrial β-oxidation capacity for 3 weeks strongly induced hepatic lipid accumulation. Similar to findings in rats [40], this did not lead to an increase in liver weight, and it is likely that a longer treatment period is necessary to obtain this NAFLD-symptom.

Visceral adipose tissue depots decreased in all analogue-treatment groups compared to control. Our results indicate that both analogues affect the distribution of body fat by mechanisms that yet have to be identified. Similar mechanisms may also be responsible for the decrease in plasma TAG by both analogues. In TTP-fed mice an increase in plasma NEFA was observed, suggesting an increased lipase-activity in the visceral adipose tissues of these animals (Table 2). The hepatic gene expression of *Cd36* was significantly increased by all three interventions, indicating a stimulation of fatty acid uptake from plasma. This suggests that both TTP and TTA can increase mobilization of fatty acids but only TTA-treated animals can efficiently metabolize these fatty acids. This effect probably added to the TTP-induced lipid accumulation in liver. TTA treatment has previously been shown to reduce lipid depots, plasma TAG and NEFA levels, and liver TAG levels through stimulated lipid catabolism in rats [22]. However, in this study liver TAG was unchanged, either due to the short treatment period or the relatively low dose of TTA utilized.

In line with a proposed block in transport of acylcarnitines across the mitochondrial membrane by TTP [15], the observed strong depletion of plasma carnitine may reflect accumulation of long-chain acylcarnitine species in the tissues off TTP-fed animals as well as TTP + TTA-fed animals. Unfortunately, we were not able to analyse acylcarnitine levels in tissues to verify this, but palmitoylcarnitine increased dramatically in plasma (Fig. 4). As mentioned above, plasma acetylcarnitine levels were almost abolished by TTP, in agreement with a block in mitochondrial β-oxidation, and thus reduced production of acetyl-CoA. It is interesting that also products of branched chain fatty acids and amino acid degradation, iso−/valerylcarnitine and propionylcarnitine, was strongly reduced by TTP, but this could be secondary to a depletion of free carnitine in these animals. Overall, this supports the use of elevated plasma long-chained acylcarnitines as an indicator of impaired mitochondrial function, and in agreement with this, high levels of palmitoylcarnitine is linked to increased risk of myocardial infarction and death in patients with chronic heart failure [6, 44]. We also show that stimulating β-oxidation with TTA increased plasma acetylcarnitine levels, probably as a result of increased acetyl-CoA production. This indicated that the Krebs cycle and ketone body production was not able to remove the surplus acetyl-CoA generated in livers of TTA-mice, despite a stimulation of *Hmgcs2* gene expression (Fig. 2). Surprisingly, plasma palmitoylcarnitine was also increased by TTA compared to control. This increase could be influenced by TTA-carnitine formation, as it was impossible to separate the TTA-carnitine peak from the palmitoylcarnitine peak (data to be published). In long-term TTA-studies with a similar TTA dose, however, the palmitoylcarnitine level was unchanged [25]. We cannot exclude that the observed increase in palmitoylcarnitine was influenced by a stimulated recruitment of fatty acids for catabolism during three weeks of TTA-treatment. In support of this, both TTA and PPARα agonists have previously been shown to increase the gene expression of *Bbox1* involved in carnitine biosynthesis [25, 45, 46], and *Bbox1* expression as well as L-carnitine was significantly increased by TTA in the current study as well as in long term studies [25]. In the TTP + TTA group, palmitoylcarnitine was insignificantly increased (Fig. 3). As palmitoyl-CoA is a substrate for ACOX1 as well as mitochondrial β-oxidation, stimulated peroxisomal activity may have partly prevented palmitoylcarnitine accumulation in plasma in these mice compared to TTP alone. Altogether, strong PPARα-agonists may complicate the use of plasma acylcarnitines to monitor mitochondrial function.

The increase in both peroxisomal β-oxidation (Fig. 2a) and number of mitochondria (Fig. 5a-b) strongly indicate that TTP and TTA co-treatment had synergetic effects on peroxisomal and mitochondrial proliferation in liver. The number of mitochondria was not significantly affected by TTA alone at this dose. The increased phospholipid level in the TTA and TTP + TTA group also supports peroxisomal proliferation, by reflecting an increase in intracellular membrane structures. In rodents, PPARα-induced peroxisome proliferation is linked to hepatic hypertrophy and hyperplasmia, resulting in increasing liver size independent of lipid accumulation [47], as seen in the TTA- and TTP + TTA treated groups in the current study. However, the observed higher number of mitochondria in livers of TTP + TTA-treated mice did not result in elevated OXPHOS capacity with octanoylcarnitine as substrate (Fig. 5). While TTA stimulated maximal respiration capacity after addition of the uncoupler FCCP, mostly due to activity at CI (Fig. 5c), TTP + TTA reduced OXPHOS (CI, CII and ETF) as well as maximum capacity, and increased gene expression of *Ucp2,* an indicator of increased oxidative stress [39]. Thus, we observed an increased mitochondrial proliferation in the co-treated animals, perhaps due to a synergistic effect on PPARα gene activation, but the mitochondria were most likely not efficient with regard to respiration and ATP-production. TTP treatment significantly reduced hepatic OXPHOS at complex I + II + ETF (Fig. 5c), indicating a link between inhibition of β-oxidation and respiration. This is in line with a recent publication demonstrating a physical connection between enzymes involved in mitochondrial fatty acid β-oxidation and OXPHOS complexes [48]. TTP did, however, not reduce the maximum capacity of oxygen consumption in uncoupled mitochondria.

Thus, the two analogues that have been studied show some similarities as activators of PPAR regulated gene expression. However, differences in their catabolism result in opposite effects on mitochondrial fatty acid oxidation and energy production, which partly modify their PPAR-dependent metabolic effects (summarized in Additional file 1: Table S1).

TTA has previously been shown to have a stronger effect on the expression of genes involved in β-oxidation in liver compared to muscle, heart and adipose tissue [26, 49, 50]. It is, however, likely that the influence of TTP and TTA on other major metabolic organs will contribute to the plasma pool of acylcarnitines, and this should be further studied. Interestingly, particularly the long and medium-chained acylcarnitines have been linked to cardiovascular risk [6, 44]. Although long-chain acylcarnitines were not increased in young individuals with obesity and type 2 diabetes [51], this phenotype is believed to be a consequence of prolonged obesity in adults [52]. In line with this, patients suffering from NASH have significantly higher levels of long-chained acylcarnitines, while they have reduced levels of short-chained acylcarnitines [53]. Our study support that this could be a result of suboptimal mitochondrial function, as inhibition of mitochondrial β-oxidation by TTP was linked to similar changes in plasma palmitoylcarnitine and acetylcarnitine.

## Conclusions

This study demonstrate how different thia-fatty acid analogues may be used to investigate disturbances in lipid metabolism and mitochondrial function related to the metabolic syndrome. TAG accumulation was accompanied by reduced OXPHOS activity in livers of TTP-treated mice, while TTA increased hepatic β-oxidation and respiratory capacity, suggesting a direct link between β-oxidation and mitochondrial respiratory efficiency in mice. Combined TTP and TTA treatment increase *in vitro* β-oxidation and reduced lipid accumulation in liver compared to TTP alone, indicating that TTA can prevent the establishment of fatty liver. However, hepatic OXPHOS capacity was lowered, suggesting dysfunctional mitochondria. Although the co-treatment with TTA was unable to rescue TTP-induced mitochondrial function in this mouse model, TTA treatment has a clear potential to improve mitochondrial function in natural situations of metabolic disease.

The study shows that plasma acylcarnitines, new markers of MI-risk in human CVD patients, reflected the reduced mitochondrial β-oxidation in TTP-fed mice, indicating that they could be useful tools to monitor mitochondrial function. The study however demonstrates that plasma acylcarnitine levels should be interpreted with caution in the presence of PPAR agonists that influence carnitine biosynthesis and turnover.

## Additional file

Additional file 1: Table S1. Overview of results. (DOCX 101 kb)

## Abbreviations

ACOX1: Acyl-CoA oxidase 1, palmitoyl
*Bbox1*: Gamma-butyrobetaine hydroxylase 1
*Cd36*: CD36 antigen/fatty acid translocase (Fat)
CI: Complex I
CII: Complex II
CoA: Coenzyme A
CPT1: Carnitine palmitoyltransferase 1
CPT2: Carnitine palmitoyltransferase 2
*Crat*: Carnitine acetyltransferase
CVD: Cardiovascular disease
*Fabp1*: Fatty acid binding protein 1, liver
MI: Myocardial infarction
NAFLD: Non-alcoholic fatty liver disease
*Ndufs1*: NADH dehydrogenase (ubiquinone) Fe-S protein 1
OXPHOS: Oxidative phosphorylation
PPARα: Peroxisome proliferator activated receptor alpha
*Sdha*: Succinate dehydrogenase complex, subunit A, flavoprotein
T2DM: Type 2 diabetes mellitus
TAG: Triacylglycerol
TEM: Transmission electron microscopy
TTA: Tetradecylthioacetic acid
TTAcr-CoA: Tetradecylthioacrylic-coenzyme A
TTP: Tetradecylthiopropionic acid
*Ucp2*: Uncoupling protein 2
