# Supplementary material for: Associations between fatty acid oxidation, hepatic mitochondrial function, and plasma acylcarnitine levels in mice
Source: Nutr Metab (Lond). 2018 Jan 29;15:10. doi: 10.1186/s12986-018-0241-7 (PMC5789604; doi:10.1186/s12986-018-0241-7)
Supplement: Additional file 1: Table S1. — Overview of results. (DOCX 101 kb) [file 12986_2018_241_MOESM1_ESM.docx]

| **Additional file 1: Table S1.** Overview of results^1^ | | | | |
| --- | --- | --- | --- | --- |
| **Parameter** | **TTP** | **TTA** | **TTA + TTP** | **Function** |
| body weight | ↓ | − | ↓ |  |
| white adipose tissue mass | ↓ | ↓ | ↓ |  |
| % adipose tissue:body weight | ↓ | ↓ | ↓ |  |
| feed intake week 1 | ↓ | − | ↓ |  |
| feed intake week 2 & 3 | − | − | − |  |
| Liver weight | ↓ | − | − |  |
| Liver:body weight | − | ↑ | ↑ |  |
| beta-ox of palmitoyl-CoA | ↓ | ↑ | ↑ | fatty acid catabolism |
| expression of *Crat* | − | ↑ | ↑↑ | carnitine acetyltransferase |
|  |  |  |  |  |
| hepatic ACOX1 activity | ↑ | ↑↑ | ↑↑↑ | peroxisomal beta-oxidation |
| expression of *Acox1* | − | ↑ | ↑ | peroxisomal beta-oxidation |
| expression of *Cpt1a* | ↑ | − | ↑ | mitochondrial beta-oxidation |
| expression of *Cpt2* | − | − | ↑ | mitochondrial beta-oxidation |
| expression of *Hmgcs2* | ↑ | ↑ | ↑ | ketone body production |
| expression of *Fabp1* in liver | − | − | − | cellular fatty acid import |
| expression of *Cd36* | ↑ | ↑↑ | ↑↑↑ | cellular fatty acid import |
|  |  |  |  |  |
| plasma level of unesterified L-carnitine | ↓↓ | ↑ | ↓ | mitochondrial transport |
| plasma level of gamma-butyrobetaine | ↓ | − | ↓ | mitochondrial transport |
| plasma level of trimethyllysine | − | − | − | mitochondrial transport |
| expression of *Bbox1* | − | ↑ | ↑ | mitochondrial transport |
| plasma level of palmityolcarnitine | ↑ | ↑ | ↑ | mitochondrial transport |
| plasma level of propionylcarnitine | ↓ | − | ↓ | mitochondrial transport |
| plasma level of iso-/valerylcarnitine | ↓ | − | ↓ | mitochondrial transport |
| plasma level of octanoylcarnitine | − | − | − | mitochondrial transport |
| plasma level of acetylcarnitine | ↓ | ↑ | ↓ | mitochondrial transport |
|  |  |  |  |  |
| TAG level in heart tissue | − | − | − | tissue lipid levels |
|  |  |  |  |  |
| hepatic TAG level | ↑↑ | − | ↑ | tissue lipid levels |
| Hepatic cholesterol level | ↑ | − | − | tissue lipid levels |
| Hepatic phospholipid level | − | ↑ | ↑ | tissue lipid levels |
|  |  |  |  |  |
| plasma TAG level | ↓ | ↓ | − | plasma lipid levels |
| plasma phospholipid level | ↓ | ↓ | − | plasma lipid levels |
| plasma cholesterol level | − | − | ↑ | plasma lipid levels |
| plasma non-esterified fatty acids | ↑ | − | − | plasma lipid levels |
|  |  |  |  |  |
| number of mitochondria | − | − | ↑ | TEM |
|  |  |  |  |  |
| OXPHOS (CI) | − | − | ↓ | OXPHOS |
| OXPHOS (CII) | − | − | ↓ | OXPHOS |
| OXPHOS (CI+CII+ETF) | ↓ | − | ↓ | OXPHOS |
| max capacity of mitochondria | − | ↑ | ↓ | OXPHOS |
| expression of *Ndufs* | − | − | − | OXPHOS |
| expression of *Sdha* | − | − | − | OXPHOS |
| expression of *Ucp2* | − | − | ↓ | OXPHOS |
| ^1^Upward pointing arrow indicates upregulation compared to control. Downward pointing arrow indicates downregulation compared to control. Dash indicates no significant change compared to control. | | | | |
